# Ovarian Transcriptome Analysis of Vitellogenic and Non-Vitellogenic Female Banana Shrimp (*Fenneropenaeus merguiensis*)

**DOI:** 10.1371/journal.pone.0164724

**Published:** 2016-10-14

**Authors:** Uraipan Saetan, Unitsa Sangket, Panchalika Deachamag, Wilaiwan Chotigeat

**Affiliations:** 1 Department of Molecular Biotechnology and Bioinformatics, Faculty of Science, Prince of Songkla University, Hat-yai, Songkhla, Thailand; 2 Center of Excellent for Genomic and Bioinformatics Research, Faculty of Science, Prince of Songkla University, Hat-yai, Songkhla, Thailand; Nazarbayev University, KAZAKHSTAN

## Abstract

The banana shrimp (*Fenneropenaeus merguiensis*) is one of the most commercially important penaeid species in the world. Its numbers are declining in the wild, leading to a loss of broodstock for farmers of the shrimp and a need for more successful breeding programs. However, the molecular mechanism of the genes involved in this shrimp’s ovarian maturation is still unclear. Consequently, we compared transcriptomic profiles of ovarian tissue from females in both the vitellogenic stage and the non-vitellogenic stage. Using RNA-Seq technology to prepare the transcriptome libraries, a total of 12,187,412 and 11,694,326 sequencing reads were acquired from the non-vitellogenic and vitellogenic stages respectively. The analysis of the differentially expressed genes identified 1,025 which were significantly differentially expressed between the two stages, of which 694 were up-regulated and 331 down-regulated. Four genes putatively involved in the ovarian maturation pathway were chosen for validation by quantitative real-time PCR (RT-qPCR). The data from this study provided information about gene expression in ovarian tissue of the banana shrimp which could be useful for a better understanding of the regulation of this species’ reproductive cycle.

## Introduction

The banana shrimp (*Fenneropenaeus merguiensis*) is widely distributed in the Indo-West Pacific region [1]. At present, the numbers and quality of broodstock sourced from wild populations are in decline because of overfishing, causing difficulties for producers of the farmed shrimp. Accordingly, improving the numbers and quality of the cultivated broodstock would be one way of addressing these issues. However, achieving this requires a better knowledge of the molecular controls on the reproductive system of this shrimp. So, in order to elucidate the molecular mechanism that regulates the system, we decided to investigate the ovarian development of *F. merguiensis* at the genomic level. The protein vitellin (Vn) is known to be a major component of the yolk in the shrimp’s eggs. It accumulates in oocytes during vitellogenesis [2] and is derived from a precursor protein, vitellogenin (Vtg) [3]. Other research has identified a number of genes involved in shrimp ovarian maturation. These include the translationally controlled tumour protein (TCTP), H-L(3)MBT-LIKE, thrombospondin (TSP), ribosomal protein L10a (RPL10a), heat shock protein 70 (HSP70), and shrimp ovarian peritrophin (SOP) [4]. But our understanding of the dynamic regulated process of the shrimp’s ovarian maturation is nevertheless still insufficient.

Transcriptomics has become a popular method of identifying genes involved in ovarian maturation and development and various studies have used different methods to analyze transcriptomes of the crustacean reproductive system. Suppression subtractive hybridization [5], differential display RT-PCR (DDRT-PCR) [6], annealing control primer [7], microarray [8], and EST-sequencing [9] have been among those employed. Some of the differentially expressed genes found by these methods in shrimp are Tra-2 [10], prostaglandin reductase 1, ubiquitin specific peptidase 9, X-linked (USP9X) [11], mitogen-activated protein kinase 1 (MAPK1) [12], guanine nucleotide-binding protein G(q) subunit alpha (Gq/11), calreticulin (CALR), calmodulin (CaM), calcineurin B (CaN), Ca2+ transporting ATPase (PMCA), guanine nucleotide-binding protein G(i) subunit alpha (G), adenylate cyclase (AC), cell division cycle25 (Cdc25), cell division cycle2 (Cdc2), cyclinB (CycB), and mitogen-activated protein kinase (MAPK) in Penaeus monodon [13], ubiquitin-conjugating enzyme E2r (UBE2r) [7], proliferating cell nuclear antigen (PCNA) [14] and heat shock protein 90 (HSP90) in *Macrobrachium nipponense* [15].

Recently, a transcriptome analysis of genes associated with reproduction and development in *F. merguiensis* was published [16] but did not touch upon a genetic comparison of the non-vitellogenic and vitellogenic stages. This study therefore aimed to identify the differentially expressed genes involved in ovarian maturation by comparing transcriptomes of the vitellogenic stage and the non-vitellogenic stage. We used next-generation high-throughput RNA sequencing technology to produce the transcriptomic libraries and the Illumina HiSeq 2000 sequencing platform provided a powerful, cost-efficient and easily reproducible tool for transcriptomics analysis [17].

## Materials and Methods

### Ethics statement

This study was approved by the Animal Ethic Committee of Prince of Songkla University.

### Sample preparation

The adult female shrimp used in this study were purchased directly from a fishing boat in Nakhon Si Thammarat Province in Thailand. Because they were selected from wild populations, the ages of the shrimp are impossible to know. Individuals were initially separated into non-vitellogenic and vitellogenic populations by observation of the ovary on their backs. After separation, they were dissected and the ovaries collected. Onset of vitellogenesis was determined by histological analysis. In the vitellogenic stage, the ovary was characterized by developing oocytes of 100-200 *μ*m showing granular yolk globules among the various small fatty droplets surrounding the nucleus [18]. The non-vitellogenic ovary, meanwhile, contained previtellogenic oocytes of < 65 *μ*m and the nucleus of each cell contained large granular nucleoli. The shrimp in the vitellogenic population weighed 60-70 g, while the non-vitellogenic shrimp weighed about 35-40 g. The shrimp were placed on ice for about 10 min prior to dissection and the collected ovaries were immediately placed in Trizol reagent (GIBCO BRL, USA). The samples of both populations were pooled from every region of the ovary; from the anterior lobe, the middle lobe and from the posterior lobe about three quarters of the way down towards the tail. They were kept at −80°C before total RNA extraction. The samples from both stages were fixed in neutral buffered formalin solution (10% formalin, 33mM NaH2PO4, 45mM Na2HPO4) for histological observation. For the preparation of each transcriptome library, total RNA was extracted from the ovaries of 3 female shrimp and pooled.

### RNA extraction and RNA sequencing (RNA-seq)

The shrimp ovarian tissue from each developmental stage was pooled and weighed. Trizol reagent (GIBCO BRL, USA) was added at the ratio of 1 mL of reagent per 100 mg of tissue, homogenized at room temperature and left for 5 min to allow complete dissociation of the nucleoprotein complexes. Chloroform 0.2 mL was added to the mixture and gently mixed by hand for 15 sec and incubated at 25°C for 3 min. The sample was centrifuged at 12,000 x g for 15 min at 4°C. After centrifugation, the upper aqueous phase was transferred to a new tube and the RNA was precipitated by adding 0.5 mL of isopropyl alcohol. This sample was then incubated at 25°C for 10 min and centrifuged at 12,000 x g for 10 min at 4°C. The precipitated RNA was obviously visible, forming a gel-like pellet at the bottom of the tube. The supernatant was removed and the RNA pellet was washed once by adding 1 mL of 75% ethanol. The sample was mixed by inverting and centrifuged at 7,500 x g for 5 min at 4°C. Finally, the RNA pellet was dried and dissolved in RNase-free water and the RNA sample was treated with DNase I to eliminate any possible DNA contaminant. The process was carried out for both sample pools.

To produce the two transcriptome libraries, the non-vitellogenic and vitellogenic samples were sequenced using the Illumina Truseq RNA Sample Preparation Kit (Illumina, San Diego, USA) at the Beijing Genomics Institute (BGI, Shenzhen, China). Briefly, using the oligo (dT) magnetic beads, the mRNA was enriched from the total RNA and then the purified mRNA was interrupted into short fragments (about 200 bp) by the fragmentation buffer. Random hexamer-primer was added to synthesize the first strand cDNA. Buffer, dNTPs, DNA polymerase I (New England Biolabs) and RNase H (Invitrogen) were added into the mixture, the second strand cDNA was synthesized, and subsequently the double stranded cDNA was purified using the Qiaquick PCR extraction kit. EB buffer was added for end repaired and Poly (A) tail addition. Finally, after ligating the sequencing adapters (Illumina PE adapters), the suitable fragments were selected for PCR amplification according to the results of agarose gel electrophoresis. The cDNA libraries were used for sequencing on an Illumina HiSeq 2000 instrument at BGI-Shenzhen, China.

### De novo assembly and functional annotation

Before further analysis, the raw sequence reads were preprocessed by eliminating the low quality reads or adapter sequences introduced during the construction of the cDNA library. The data was then further cleaned by using the SOAPnuke program to remove reads containing more than 10% of unknown bases (N) and those defined as low quality because more than 50% of their bases had a quality value ≤ 5. The sequence reads of only the non-vitellogenic library were de novo assembled using Trinity RNA-seq software (version 2.0), then SOAPaligner/SOAP2 was used to map the clean reads to the reference genome. The contigs and singletons were normally referred as unigenes. The functional annotation of unigenes, which provides the information of protein function, Clusters of Orthologous Group (COG) functional annotation and Gene Ontology (GO) functional annotation were first analyzed via blastx against protein databases such as NCBI non-redundant (Nr) protein database, Swiss-Prot, KEGG and finally COG database (e-value < 0.00001). Then, they were aligned to the nucleotide database NT (e-value < 0.00001) via blastn. The Blast2GO (version 2.2.5) program was used to get GO annotation. Following this step, WEGO software was used to obtain the GO functional classifications and distribution of the genes. To refine further our understanding of the biological functions of a particular gene, the unigenes was assigned to KEGG pathway analysis by the online KEGG Automatic Annotation Server (KAAS) (http://www.genome.jp/kegg/kaas/).

### Screening of differentially expressed genes (DEGs)

To identify the DEGs between the 2 samples, the false discovery rate (FDR) method was used to determine a threshold value of p-. The FDR value was ≤ 0.001, the p-value was ≤ 0.001. A level change greater than 2 fold and an absolute value of log2Ratio(vitellogenic stage RPKM/non-vitellogenic stage RPKM) ≥ 1 were used to judge the significance of the gene expression differences. NOISeq was used to screen the differentially expressed genes of the vitellogenic and non-vitellogenic ovaries. In addition, RPKM (reads per kilobase per million reads) is a method of quantifying the gene expression from RNA sequencing data by normalizing the total read length and the number of mappable reads [19].

### Confirmation analysis of marker genes expression involved in ovarian maturation

Various genes have to date been reported involved in ovarian maturation. These include Vn and several others shown in Table 1. To confirm the quality of the output data from the RNA-seq protocol, bioinformatics programs were used to verify the presence of some of these genes. Selected marker sequences were aligned with RNA sequences data from assembled libraries by running a BLAST search.

**Table 1:** Marker genes expression involved in ovarian maturation.

Gene	Accession
TCTP	AAV84282
SOP	AAV83539
RPL10a	ACU52718
TSP	ACV32380
Vtg	ACV32381
L(3)MBT-like protein 3-like	XP_ 792284 XP_ 002190400 XP_ 001606620
HSP70	CAL68995 AFX84616 AEE81035 ACN38704 ABV46675 AAT46566 CAL68992 BAJ78982 ABF85672

### Validation of gene expression by RT-qPCR analysis

In the functional annotation, the KEGG pathway analysis identified the involvement of eleven specific pathways in ovarian maturation including crustacean calcium binding protein (CBP), insulin-like receptor-like (InR), and 5-hydroxytryptamine receptor 2B-like isoform 1 (5-HT2B). These three were chosen for the analysis of gene expression by RT-qPCR. Total RNA was extracted from both ovarian stages as described in the transcriptome profiling section. Nine shrimp in the non-vitellogenic stage and nine in the vitellogenic stage were used for the validation. They were divided into three replicates; each replicate was extracted for RNA sample and then pooled together. Each RNA pooled sample was amplified in three technical replicates.

To synthesize cDNA from RNA, 2 *μ*g of total RNA were mixed with 100 ng of random primer, incubated at 70°C for 5 min and immediately placed on ice for 5 min to denature the RNA. Then, the RNA was mixed with buffer comprising RT buffer, 10 mM dNTP, AMV reverse transcriptase (0.2 units/*μ*l) and treated with DEPC water. Finally, the mixture was incubated at 48°C for 2 h to convert it to cDNA. The RT-qPCR reaction contained 300 ng of cDNA, 20 pmol each of forward and reverse primer, 1x FastStart Universal SYBR Green Master (Roche, Germany). The volume of the reaction was adjusted to 50 *μ*l with deionized water. The PCR cycling began with a denaturation step at 95°C for 5 min, followed by 40 cycles of 94°C for 30 sec, annealing at the required temperature for 30 sec and finally extension at 72°C for 45 sec. Beta-actin (*β*- actin) was used as an endogenous control. Thermal cycling and fluorescence detection were conducted using the Mx3000PTM (Stratagene, CA, USA). To quantify the expression of all genes, the standard curve was prepared using 10 fold serial dilutions of linearized purified PCR products. The dynamic range of detection was in between 1X 10^2^ and 1X 10^6^ copies. The ratio of the copy number of the interested gene to the copy number of the *β*-actin gene in each sample was calculated. Data was expressed as mean ± SD.

## Results and Discussion

### Transcriptome sequencing data

To obtain information about the ovary transcriptome of *F. merguiensis* and analyze the genes involved in ovarian maturation of the species, two cDNA libraries were constructed from ovarian tissue sampled from individuals in both the non-vitellogenic stage and the vitellogenic stage of development. The Illumina HiSeq 2000 sequencing platform generated more than 12 million reads from the non-vitellogenic stage and over 11 million reads from the vitellogenic stage. The raw sequence data were deposited in the NCBI Sequence Read Archive (SRA) under accession number SRP075844. After the low quality reads and short reads were trimmed, approximately 99% of the raw reads completely passed the filter with 12,187,412 non-vitellogenic and 11,694,326 vitellogenic clean reads obtained. In addition, the total generated base pairs were 597,183,188 non-vitellogenic and 573,021,974 vitellogenic (Table 2). The de novo assembly output found 41,877 and 62,114 with N50 of these reads, 1533 were unigenes and 1084 were contigs. In the functional annotation analysis, unigenes were annotated to the NR, NT, Swiss-Prot, KEGG, COG and Go databases. In the protein coding region analysis, the total number of CDS was 28,857 and the number of CDS that mapped to the protein database was 16,677 (Table 3).

**Table 2:** Statistical alignment results of non-vitellogenic and vitellogenic libraries mapped to reference genome.

Sample	Non-vitellogenic	Vitellogenic
Total Reads	12,187,412 (100.00%)	11,694,326 (100.00%)
Total Base Pairs	597,183,188 (100.00%)	573,021,974 (100.00%)
Total Mapped Reads	11,006,273 (90.31%)	10,715,709 (91.63%)
Perfect Match	8,522,152 (69.93%)	8,516,236 (72.82%)
≤ 2bp Mismatch	2,484,121 (20.38%)	2,199,473 (18.81%)
Unique Match	6,888,930 (56.52%)	6,759,393 (57.80%)
Multi-position Match	4,117,343 (33.78%)	3,956,316 (33.83%)
Total Unmapped Reads	1,181,139 (9.69%)	978,617 (8.37%)

**Table 3:** Statistics of de novo assembly of non-vitellogenic shrimp transcriptome.

**Assembly quality**	
Total contig number with N50	62,114 with 1,084
Total unigene number with N50	41,877 with 1,533
**Functional annotation**	
Number of sequences with BLAST (nr)	16,460
Number of sequences with NT	8,451
Number of sequences with Swiss-Prot	14,556
Number of sequences with KEGG	12,459
Number of sequences with COG	6,611
Number of sequences with GO	8,433
Total annotation number of unigenes	18,826
**Protein coding region prediction**	
Number of CDS mapped to protein database	16,677
Number of predicted CDS	12,180
Total number of CDS	28,857

### Sequencing saturation analysis

**Fig 1:**
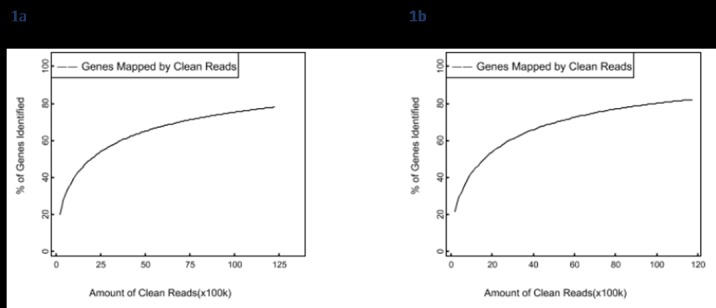
Sequencing saturation analysis. The X-axis represents the number of clean reads and Y-axis the percentage of identified genes. Fig 1a shows the DEGs of the non-vitellogenic stage sample and 1b the DEGs of the vitellogenic.

To verify that the amount of discovered genes increased proportionally to the sequencing quantity, a sequencing saturation analysis was executed. This analysis was used to determine the sequencing data of each sample. The augmentation of the amount of reads also revealed that the amount of discovered genes augmented. Nevertheless, if the amount of reads achieves a certain value, the growth rate of discovered genes should flatten and saturate. Fig 1 illustrates the trend of saturation when the amount of discovered genes still increases.

**Fig 2:**
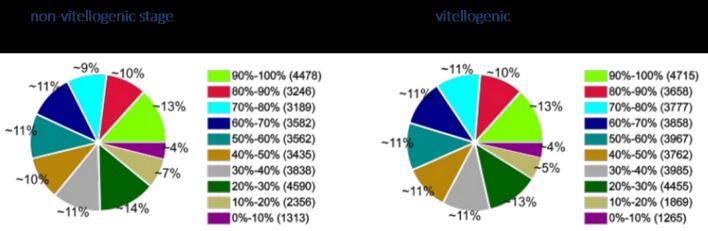
Distribution of gene coverage identification for DEGs.

Gene coverage is the percentage of a gene covered by reads. The value is equal to the ratio of total base count in a gene covered by unique mapping reads to the total base count for that gene. Gene coverage was estimated for both libraries as shown in Fig 2. The data shows the distribution gene coverage derived from reads of both libraries. Approximately 10% of the total genes in both had coverage between 80-90% (3,246 and 3,658 in non-vitellogenic and vitellogenic libraries respectively) and about 13% of the total genes had 90-100% gene coverage (4,478 and 4,715), suggesting that the read distribution and quality of read mapping are similar between the two libraries.

### Identification and analysis of DEGs involved in ovarian maturation

**Fig 3:**
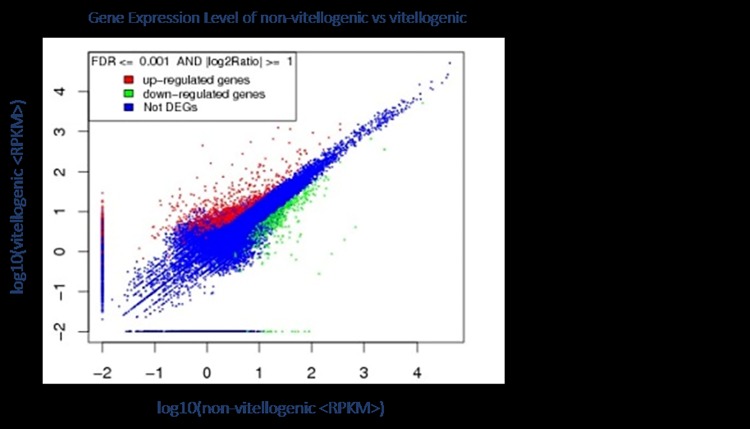
Comparison of gene expression levels between vitellogenic and non-vitellogenic stages. Red spots represent up-regulated genes, and green spots indicate down-regulated genes. Blue spots represent genes that did not show obvious changes between vitellogenic and non-vitellogenic stages.

To discover the genes differentially expressed between the non-vitellogenic stage and the vitellogenic stage, the gene expression levels were calculated using the RPKM method (Reads per kb per million mapped reads). To classify the significance of each gene expression difference, false positives in the DEGs were determined by false discovery rate (FDR) any using the threshold FDR ≤ 0.001. The absolute value of log2Ratio ≥ 1 signified up-regulation while log2Ratio ≤ 1 showed down-regulation. In total, 1,025 genes were significantly differentially expressed, composed of 694 genes identified up-regulated and 331 down-regulated between the two libraries (Fig 3).

The identified up-regulated marker genes expressed during vitellogenesis are shown in Table 1

### Functional annotation of DEGs

**Fig 4:**
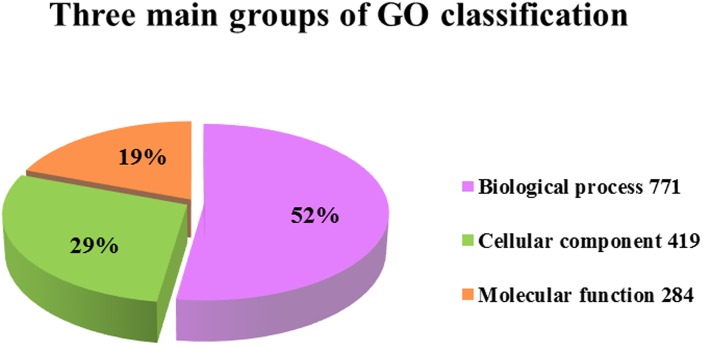
Three main groups of GO classification. Pie diagrams of the ontological distribution of sequences by percentage for all DEGs.

The ontological functions of the DEGs were determined by mapping to GO terms. A total of 1,025 genes were ontologically classified. Some genes were classified in more than one domain giving 1,474 genes classifications. The domain most represented was biological process with 771 mapped DEGs (52.3%) via both cellular process and metabolic process. The next most represented domain was cellular component with 419 DEGs (28.4%) via cell and cell part and the least represented domain was molecular function with 284 mapped DEGs (19.3%) via catalytic activity and binding (Fig 4).

The pathway analysis of the DEGs was obtained from searching the KEGG pathway database which assigned 519 genes to 214 biological pathways. The largest pathway category was the metabolic pathway with 64 annotated genes representing 12.33% of the total. However, only eleven specific pathways involved in ovarian maturation were observed. These were steroid biosynthesis, serotonergic synapse, insulin signaling pathway, MAPK signaling pathway (fly), GnRH signaling pathway, steroid hormone biosynthesis, calcium signaling pathway, progesterone-mediated oocyte maturation, ribosome, MAPK signaling pathway and ubiquitin mediated proteolysis. We examined several of the hormones and genes implicated in the regulation of the crustacean reproductive process (Table 4). Having considered the pathways involved in ovarian maturation, we selected four genes for validation of gene expression by RT-qPCR: crustacean calcium binding protein (CBP), insulin-like receptor-like (InR), 5-hydroxytryptamine receptor 2B-like isoform 1 (5-HT2B), and ribosomal protein L10a (RPL10a).

**Table 4:** Putative candidate genes involved in crustacean ovarian maturation.

Gene	log2Ratio (vitellogenic/non-vitellogenic)	Up-Down regulation	P-value
Calnexin	0.849779348	Up	7.93E-10
CBN-XNP-1 protein	−1.88684786	Down	4.30E-06
Crustacean calcium-binding protein 23	1.791187916	Up	4.35E-106
Crustacean hyperglycemic hormone, putative	3.37791951	Up	4.08E-11
Farnesoic acid O-methyltransferase	1.652938862	Up	1.27E-116
Growth hormone-inducible soluble protein, putative	−0.76215796	Down	3.57E-04
Growth hormone-inducible transmembrane protein isoform 2	−0.70150601	Down	5.26E-05
Heat shock protein 90	0.623246273	Up	2.55E-234
JHE-like carboxylesterase 1	2.695106789	Up	1.78E-250
MAPK kinase 1 interacting protein 1	−0.46125938	Down	9.29E-05
Neuroparsin 1 precursor	3.446961154	Up	2.91E-43
PREDICTED: 5-hydroxytryptamine receptor 2B-like isoform 1	5.453687017	Up	1.73E-12
PREDICTED: insulin-like growth factor 1 receptor-like	0.889494355	Up	4.23E-11
PREDICTED: insulin-like receptor-like	1.414445386	Up	7.75E-06
PREDICTED: proliferation-associated protein 2G4-like	0.186983881	Up	1.47E-10
PREDICTED: receptor for egg jelly 7-like	1.546796422	Up	1.53E-04
Progestin membrane receptor component 1	0.188218475	Up	1.51E-01
Thyroid hormone receptor interactor 10	1.072746253	Up	1.66E-05
Thyroid hormone receptor-associated protein, putative	0.782103445	Up	2.09E-12
VASA	0.263310527	Up	6.61E-07
Vitellogenin receptor	0.861881603	Up	5.77E-247

### DEGs involved in ovarian maturation

The ovarian development and maturation processes of shrimp reproduction are controlled by several important factors, such as neurotransmitters, various hormones and their receptors [20]. From the transcriptome analysis, the proliferation process of the shrimp’s ovary involves several pathways including the serotonergic synapse, the insulin signaling pathway, the GnRH signaling pathway and the calcium signaling pathway. From among these pathways, candidate genes were examined for their role in ovarian maturation. We focused our attention on the serotonergic synapse and insulin signaling pathways associated with ovarian maturation. The KEGG pathway analysis showed the presence of 5-HT2B in the serotonergic synapse pathway. This is a receptor of serotonin (5-HT), which is a monoamine neurotransmitter that plays an important role in physiological function and endocrine secretion in both vertebrates and invertebrates. Several studies have reported the function of 5-HT in ovarian development and maturation in many shrimp species, including *P. monodon* [21, 22], *P. vannamei* [23], *M. nipponense* [24], *M. rosenbergii* [25, 26] and *F. merguiensis* [27].

Several studies of the insulin signaling pathway have shown the involvement of insulin in ovarian maturation resulting in mutation of the *Drosophila* insulin receptor and CHICO in smaller ovaries [28]. Insulin also functions to regulate oogenesis and ovarian maturation in other organisms such as the rat, in which insulin is an endocrine factor of primary follicle transition in oogenesis [29]; it is also present in the ovaries of the carp [30]. Moreover, the insulin signaling pathway also includes calcium binding protein (CBP), a heterogeneous and wide group of proteins that partake in various cellular functions such as homeostasis and cell signaling [31]. A previous study found a connection in the central nervous system of *M. rosenbergii* and *P. monodon* between CBP and gonadotrophin-releasing hormone (GnRH), a neuropeptide known to be involved in the stimulation of oocyte maturation [20].

This transcriptome analysis found that the vitellogenin receptor (Vtgr) gene up-regulated significantly higher in the vitellogenic stage of the ovary’s development than in the non-vitellogenic stage, further evidence supporting this gene’s involvement in ovarian maturation. Earlier research, reporting a significantly higher expression level of Vtgr in broodstock at the late stage of ovarian development of *P. monodon* than in juveniles, revealed the involvement of this gene in oogenesis [32]. Another important gene in shrimp reproduction is progestin membrane receptor component 1 (Pgmrc1) which was recently reported to stimulate ovarian maturation. Pgmrc1 is a putative membrane bound receptor of progestin that may play a crucial role in gametogenesis [33] and act to induce the meiotic maturation of the oocyte [34]. A study of *P. monodon* found a higher expression level of Pgmrc1 in the ovaries of broodstock than of juveniles and also found Pgmrc1 protein in the follicular layer and cell membrane of follicular cells at several stages of oocytogenesis [35]. This transcriptome analysis found that Pgmrc1 was highly up-regulated in vitellogenic shrimp which correlated with previous reports, implying that it may play a vital role in shrimp ovarian maturation.

### RT-qPCR validation

The expression patterns of four genes were tested by using RT-qPCR to validate the RNA-seq results. The expression patterns of the four genes and the control gene were consistent with the RNA-seq results (Table 5), which suggests that the results of the RNA-seq experiments were accurate and the experimental design efficient. Although several studies had reported the function of 5-HT in ovarian development and maturation, the mechanism of 5-HT was nevertheless still unclear until the study of the injection of 5-HT into female shrimp [36]. This detected an increase in the level of methyl farnesoate (MF) in the hemolymph, suggesting that 5-HT is involved in regulating the release of MF in shrimp via the thoracic ganglion. The finding that 5-HT2B, which is a receptor of 5-HT, was up-regulated more in the vitellogenic stage of the shrimp ovary than in the non-vitellogenic stage may point to its direct involvement in ovarian development and maturation.

**Table 5:** Validation of the expression profile between RNA-Seq and RT-qPCR for selected genes.

Gene	Pathway	(vitellogenic/non-vitellogenic)		Primer sequences
		RNA-seq	RT-qPCR	
CBP	Calcium signaling pathway GnRH signaling pathway Insulin signaling pathway	3.40	9.74	F: GAGAAGCTGAGGAACCAC R: CTTGTCCAGGTGCTTGAA
InR	Insulin signaling pathway	2.62	8.77	F: CGAAACGTGCCCTCCT R: TTCCAGTCCTCTTCGGTT
5-HT2B	Calcium signaling pathway Serotonergic synapse	43	2.34	F: CCTCGCTGTCTCGGATT R: CTTTGGCCGTACTGGAT
RpL10a		1.03	3.18	F: GCGTTTCAGTGGCACAGTG R: TTCTGCCAATGCTTCTTCAAC
*β*-actin				F: CAGATCATGTTYGAGACCTTC R: GATGTCCTCGTCRCACTTCAT

During the ovarian development and maturation of shrimp, ribosomal protein L10a (RpL10a) is one of the genes that up-regulates in the late vitellogenic stage [4]. A relation between RpL10a and insulin in *D. melanogaster* was found in a previous study in which RpL10Ab- germline clones were mutated, resulting in the premature death of follicle cells similar to insulin receptor mutants [37]. The *in silico* analysis had the RpL10A protein directly binding to the insulin receptor at a different site than the insulin molecule. This may indicate that the insulin receptor’s interaction was prolonged by the presence of RpL10A (submitted data). In this study, the expression of insulin-like receptor-like (InR) and RpL10a was up-regulated in the vitellogenic stage which is correlated with transcriptome data of a link between the insulin receptor and RpL10a. It also accords with a previous report that the insulin receptor may regulate the reproductive function of ovaries in mice [38]. In addition, CBP, which participates in the insulin signaling pathway, also up-regulated in this study. The calcium binding protein calmodulin affects calcium levels in the GnRH pathway regulating oocyte maturation in many organisms, including the rat. The calcium-calmodulin system participates in the gonadotropic regulation of granulosa cell steroidogenesis independently of cellular differentiation [39], consistent with findings showing an involvement of calmodulin in ovarian steroid production in teleost fish, Atlantic croaker (*Micropogonias undulates*) [40] and crustaceans [41]. More evidence of calmodulin’s effect on oocyte maturation was found in *Xenopus*, which shows no significant response to calmodulin injection [42], and in *Rana pipiens* [43]. In summary, all of these data suggest that various DEGs are possibly crucial in the regulating of ovarian development and maturation in shrimp.

## Conclusions

This study investigated the transcriptomic profiles of ovarian tissue from banana shrimp in the non-vitellogenic stage and the vitellogenic stage of development using RNA-Seq technology. In total, we found 1,025 differentially expressed genes among the two libraries we constructed from the two populations. From the KEGG analysis, eleven pathways were classified as having involvement in ovarian maturation. The differential expression of some of the genes in these pathways was confirmed by RT-qPCR. From this study, various data were collected which could prove valuable for a more comprehensive knowledge of the reproductive system of this shrimp during the ovarian maturation process.
